# Heterogeneous *Klebsiella pneumoniae* Co-infections Complicate Personalized Bacteriophage Therapy

**DOI:** 10.3389/fcimb.2020.608402

**Published:** 2021-01-25

**Authors:** Jinhong Qin, Nannan Wu, Juan Bao, Xin Shi, Hongyu Ou, Shanke Ye, Wei Zhao, Zhenquan Wei, Jinfeng Cai, Lisha Li, Mingquan Guo, Jingyan Weng, Hongzhou Lu, Demeng Tan, Jianzhong Zhang, Qin Huang, Zhaoqin Zhu, Yejing Shi, Chunlan Hu, Xiaokui Guo, Tongyu Zhu

**Affiliations:** ^1^ Department of Microbiology and Immunology, Shanghai Jiao Tong University School of Medicine, Shanghai, China; ^2^ Shanghai Institute of Phage, Shanghai Public Health Clinical Center, Fudan University, Shanghai, China; ^3^ School of Global Health, Chinese Center for Tropical Diseases Research, Shanghai Jiao Tong University School of Medicine, Shanghai, China; ^4^ Department of Urology, Shanghai Public Health Clinical Center, Fudan University, Shanghai, China; ^5^ School of Life Sciences & Biotechnology, Shanghai Jiao Tong University, Shanghai, China; ^6^ Department of Infectious Diseases, Shanghai Public Health Clinical Center, Fudan University, Shanghai, China; ^7^ Experiment Teaching Center of Basic Medicine, Shanghai Jiao Tong University School of Medicine, Shanghai, China; ^8^ Core Facility of Basic Medical Sciences, Shanghai Jiao Tong University School of Medicine, Shanghai, China; ^9^ Department of Laboratory Medicine, Shanghai Public Health Clinical Center, Fudan University, Shanghai, China; ^10^ Department of Microbiology, School of Basic Medical Science, Guizhou Medical University, Guiyang, China; ^11^ Department of Pharmacy, Zhongshan Hospital, Fudan University, Shanghai, China; ^12^ Shanghai Key Laboratory of Organ Transplantation, Zhongshan Hospital, Fudan University, Shanghai, China

**Keywords:** urinary tract infection, phage therapy, percutaneous nephrostomy, heterogeneous cells, multidrug-resistant *Klebsiella pneumoniae*

## Abstract

Multidrug-resistant (MDR) organisms have increased worldwide, posing a major challenge for the clinical management of infection. Bacteriophage is expected as potential effective therapeutic agents for difficult-to-treat infections. When performing bacteriophage therapy, the susceptibility of lytic bacteriophage to the target bacteria is selected by laboratory isolate from patients. The presence of a subpopulation in a main population of tested cells, coupled with the rapid development of phage-resistant populations, will make bacteriophage therapy ineffective. We aimed to treat a man with multifocal urinary tract infections of MDR *Klebsiella pneumoniae* by phage therapy. However, the presence of polyclonal co-infectious cells in his renal pelvis and bladder led to the failure of three consecutive phage therapies. After analysis, the patient was performed with percutaneous nephrostomy (PCN). A cocktail of bacteriophages was selected for activity against all 21 heterogeneous isolates and irrigated simultaneously *via* the kidney and bladder to eradicate multifocal colonization, combined with antibiotic treatment. Finally, the patient recovered with an obviously improved bladder. The success of this case provides valuable treatment ideas and solutions for phage treatment of complex infections.

**Clinical Trial Registration:**

www.chictr.org.cn, identifier ChiCTR1900020989.

## Introduction

Urinary tract infections (UTIs) are among the most prevalent microbial diseases in both men and women during their lifespan and cause a major burden worldwide (Sihra et al., 2018). Administration of antibiotics is one of the key means for the management of infectious pathogens. However, MDR organisms have increased worldwide, posing a major challenge for the clinical management of infection (Moellering, 2010). One of the most urgent areas is the rapid evolution of antibiotic resistance among *Enterobacteriaceae* e.g., *Klebsiella pneumoniae* (*K. pneumoniae*) (Tacconelli et al., 2018). MDR *K. pneumoniae* is known to cause various bodily infections, including UTIs, pneumoniae, bloodstream infections, and sepsis. It is also a threat to individuals with weak immune systems and hospitalized patients following invasive surgical procedures. Infections caused by these organisms are not only difficult to treat but are also known to cause significant mortality (Marr and Russo, 2019). Bacteriophage is expected as potential effective therapeutic agents for difficult-to-treat infections, with some successful case reports supported by a large fundamental knowledge base (Schooley et al., 2017; Watts, 2017; Corbellino et al., 2019; Dedrick et al., 2019; Schmidt, 2019).

Heteroresistance phenomena was first described in the 1940s (Alexander and Leidy, 1947), which refers to seemingly identical bacterial cells in a population with one subpopulation or several subpopulations that exhibit increased levels of antibiotic resistance compared with the main population (Andersson et al., 2019). Such populations are often difficult to detect and cause antibiotic treatment failure (Nicoloff et al., 2019). Phage therapy also requires phage screening with clinically isolated strains to select the appropriate phage for treatment. Due to the high specificity of bacteriophage, the presence of bacterial heterogeneity in a population can also lead to the failure of bacteriophage therapy. Thus, a phages that will be selected for treatment should have a broad range of activity. In the present study, we report a case of polyclonal heterogenous bacterial UTI treated by personalized bacteriophage cocktails. After four round phage screenings against clinical isolates, bacteriophage cocktail with combination of antibiotics and PCN eventually cured the patient of long-term MDR *K. pneumoniae* UTI.

## Materials and Methods

### Bacteriological Studies

Bacterial isolates were obtained from routine microbiological cultures from patient. Twenty-one *K. pneumoniae* strains were recovered from the patient’s urine, renal pelvis effusion and proximal ureteral stent tip, as detailed in **Table 1**. Identification of isolates at the species level was obtained by MALDI-TOF Biotyper (Bruker, Germany).

**Table 1:** *Klebsiella pneumoniae* strains and their phage susceptibilities.

Strain	Date	Origin	Phage sensitive				
			ФJD902	ФJD905	ФJD907	ФJD908	ФJD910
4137	25 Nov 2017	Urine	+	+	+	+	+
0344	8 Jan 2018	Urine	+	+	+	+	+
1231	26 Jan 2018	Urine	+	+	+	+	+
1280	28 Jan 2018	Urine	+	+	+	+	+
1439	30 Jan 2018	kidney^r^	–	+	–	+	+
1440	30 Jan 2018	kidney^l^	+	+	+	+	+
1469	31 Jan 2018	Urine	+	+	+	+	+
1518	1 Feb 2018	Urine	–	+	+	+	+
1532	2 Feb 2018	Urine	–	+	+	+	+
1591	3 Feb 2018	Urine	+	+	+	+	+
1639	4 Feb 2018	Urine	+	+	+	+	+
1667	5 Feb 2018	Urine	–	+	+	+	+
1769	6 Feb 2018	Urine	–	+	–	+	+
1789	7 Feb 2018	Urine	–	+	+	+	+
3549	21 Mar 2018	Double J ^b^	+	+	–	+	+
3637	21 Mar 2018	Double J ^k^	+	+	–	+	+
3837	26 Mar 2018	Urine	+	+	–	+	+
4078	28 Mar 2018	Urine	+	+	+	+	+
4163	1 Apr 2018	Urine	+	+	+	+	+
4247	3 Apr 2018	Urine	+	+	–	+	–
4321	4 Apr 2018	Urine	+	+	+	+	+

Kidney^r^ represents right renal pelvis effusion; kidney^l^ represents left renal pelvis effusion; Double J ^b^ represents stent tip from bladder; Double J ^k^ represents stent tip from right renal pelvis. “+” represents lytic activity; “-” represents non-lytic.

Minimum inhibitory concentrations (MICs) for carbapenems (imipenem and ertapenem) and aminoglycosides (gentamicin, amikacin and tobramycin) were determined using VITEK 2 COMPACT (bioMérieux), and tigecycline was determined using E-test strips (Oxoid) on Mueller-Hinton agar plates (Oxoid). The MICs for colistin were determined by broth culture microdilution. Meropenem (Oxoid) was determined using the disc diffusion test on Mueller-Hinton agar plates (Oxoid). The results were interpreted according to the CLSI2018 (Clinical and Laboratory Standards Institute).

### Illumina WGS and Phylogenetic Analysis

The clonal relationship of the isolates was analysed by whole-genome sequencing. Briefly, total DNA from the *K. pneumoniae* isolates was extracted and sequenced with the Illumina X10 (Illumina, San Diego, CA, USA). Genome assembly was performed using the Velvet 1.0.15 program (Zerbino and Birney, 2008). The sequence of *K. pneumoniae* was deposited in the GenBank databases with accession number (SAMN13324145, SAMN13324146, SAMN13324147, SAMN13324148, SAMN13324149, SAMN13324150, SAMN1332415, SAMN13324152, SAMN13324153, SAMN13324154, SAMN13324155, SAMN13324156, SAMN13324157, SAMN13324158, SAMN13324159, SAMN13324160, SAMN13324161, SAMN13324162, SAMN13324163, SAMN13324164, SAMN13324165). Genome-wide single nucleotide polymorphism (SNP) calling and phylogenetic analysis were performed by using kSNP v3 (Gardner et al., 2015). The genome sequences of 4 K*. pneumoniae* ST15 isolates, including PMK1, BR, Kp-Geo-39795 and Kp36, were downloaded from GenBank. The phylogeny scheme was generated from the kSNP3-detected SNP sites for all the genome sequences under analysis with k = 21, as determined by Kchooser. A parsimony tree was generated by kSNP3 based on an extended majority rule consensus of the equally most parsimonious trees from a sample of 100 trees. The tree was displayed with iTOL with midpoint rooting (Letunic and Bork, 2016).

### Bacteriophage Studies

Bacteriophages were purified by caesium chloride (CsCl) density-gradient ultracentrifugation. Transmission electron microscopy (Hitachi 700, Tokyo, Japan) studies were performed on the bacteriophage preparation after staining with 2% phosphotungstic acid. Bacteriophage DNA was extracted with the Phage DNA Isolation Kit (Aidlab Biotech, Beijing). PacBio single-molecule real-time (SMRT) sequencing was performed using a PacBio RSII sequencer with C4 chemistry. De novo assembly was conducted using the Hierarchical Genome Assembly Process (HGAP) method based on the SMRT Analysis package 2.0. All of the ORFs predicted by Prokka (Seemann, 2014). Using BLAST at the NCBI, comparative genome analysis of phage was carried out. The prediction of the conserved protein domain was conducted using BLASTP and the NCBI Conserved Domain Database. The sequence of bacteriophages has been deposited in the GenBank databases with accession number (SAMN13324166, SAMN13324167, SAMN13324168, SAMN13324169, SAMN13324170).

### The MDR *K. pneumoniae* Infection and Pathophysiology of the Patient

**Figure 1:**
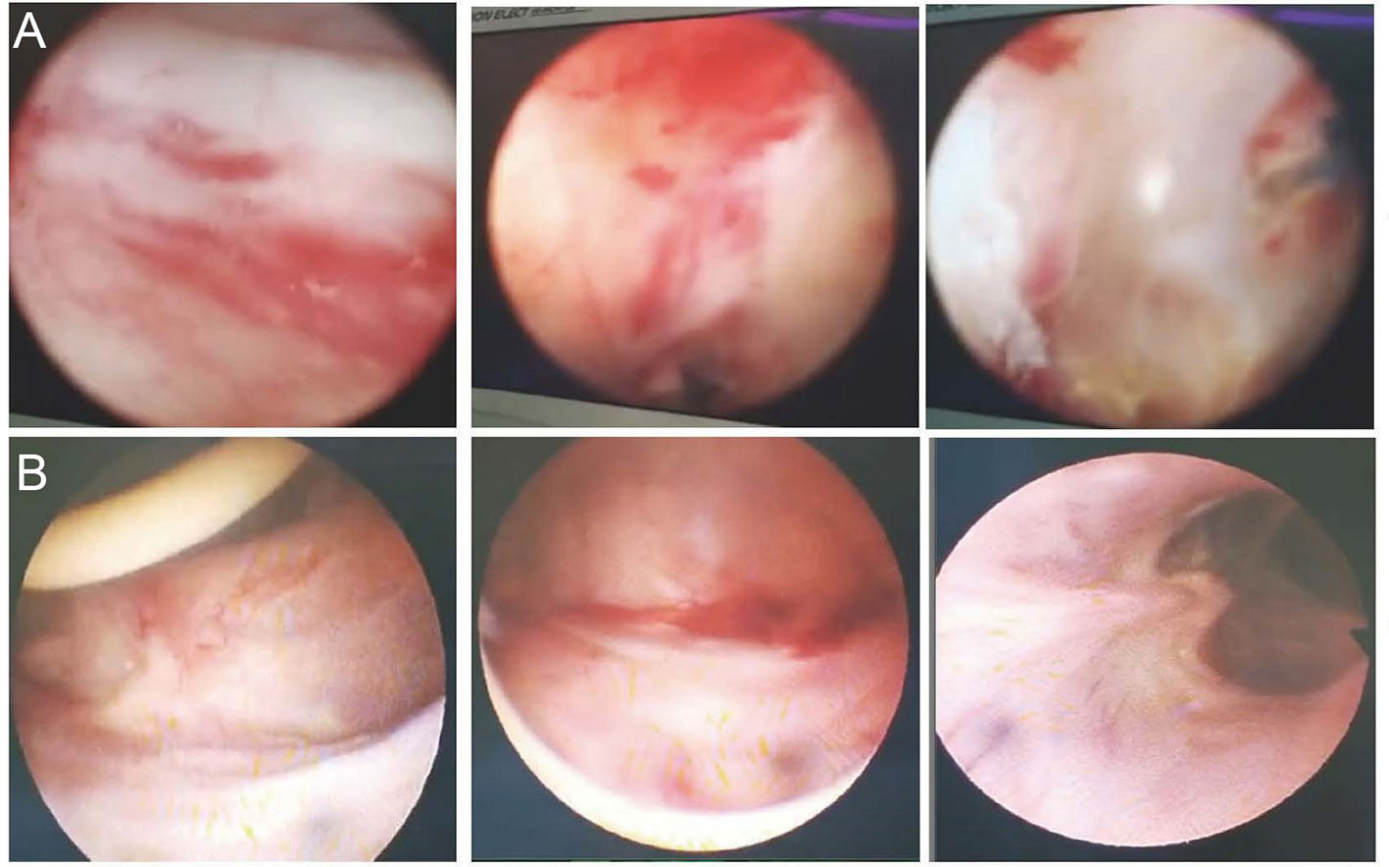
Cystoscope of the inner wall of the bladder. **(A)** The inner wall of the bladder before phage treatment. The bladder mucosa was hyperemic with local ulceration and pseudomembrane attachment; bilateral ureteral openings were not clearly observed. **(B)** The inner wall of the bladder post phage treatment. Bladder mucosa was smooth and complete; bilateral ureter openings were clear.

A 66-year-old man whose cancerous bladder was partially excised in 2002 was enrolled. He had UTIs since 2006. MDR *K. pneumoniae* was the causative agent that led to frequent and urgent urination and dysuria over the past dozen years. Antibiotics that have *in vitro* activity against the isolates have been used for conventional treatment since then. However, none of those antibiotics or their combination worked to eradicate the pathogen. The UTIs with *K. pneumoniae* reappeared immediately post drug withdrawal and were continuously susceptible to the previous antibiotic panels. A cystoscope scan showed that his bladder mucosa was hyperemic with local ulceration and pseudomembrane attachment; bilateral ureteral openings were not clearly observed (**Figure 1A**). He was recruited with hospital admission to receive bacteriophage treatment at the Shanghai Public Health Clinical Center (ChiCTR1900020989), Shanghai, China.

### Selection and Preparation of Therapeutic Bacteriophages

Bacteriophage collections were stored at Shanghai Public Health Clinical Center, which was isolated from various environmental samples by using routine isolation techniques (Wommack et al., 2009). Bacteriophages used for this treatment were screened from bacteriophage collection. The lytic activity of the bacteriophage was screened *via* spot testing against successive patient isolates as those isolates became available. To evaluate the killing efficacy of each phage on clinical isolates, 1 μl dilution aliquots of 10-fold serial dilutions of each bacteriophage was spotted on a bacterial lawn to observe plaque formation. The bacteriophage candidates that showed the strongest antibacterial activity and broad spectrum against available isolates as measured by this assay were selected for inclusion in the therapy. Bacteriophages were generated using solid media and recovered by diffusion into SM buffer (5.8 g/L NaCl, 20 mM Tris HCl pH 7.5, 2 g/L mM MgSO4.7H_2_O), yielding lysates with titres of >1x10^10^ pfu/ml. These lysates were concentrated using CIM^®^ Anion-exchange column QA (BIA Separations, Slovenia) according to the protocol. The concentration was dialysed against 0.9% sodium chloride physiological solution (Shandong Qidu Pharmaceutical Co., Ltd.). The resulting lysate was further sterilized through 0.22 μm filters. The final bacteriophage preparation was used for therapeutic application, with a titre estimated at >5x10^9^ pfu/ml.

### Bacteriophage Therapy

**Figure 2:**
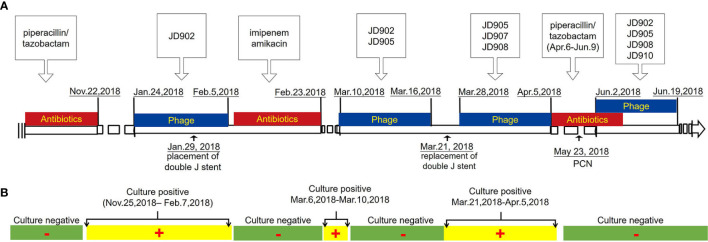
Time course of clinical treatment. **(A)** Timeline beginning with patient hospitalization and ending with recovery. Major treatment during the course is indicated above the line. Surgery during the course is indicated below the line. **(B)** Laboratory culture event and culture result during the course.

The entire treatment process for the patient is shown in **Figure 2A**. Strain *Kp*0344 were used as host to amplify bacteriophage Ф902, ФJD908, and ФJD910. Strain *Kp*1440 were used as host to amplify bacteriophage Ф905 and ФJD907. Fifty ml of the bacteriophage preparation containing 5×10^8^ pfu/ml was irrigated *via* bladder every 48 h for 2 weeks at a time. For the fourth bacteriophage treatment, in addition to irrigation *via* the bladder, 10 ml of the phage preparation containing 5x10^8^ pfu/ml was also irrigated *via* the kidney every 48 h for 2 weeks. Prior to the fourth phage treatment, we performed a bilateral PCN on the patient (**Figure S1**). The patient was hospitalized during therapy. The clinical examination and urine culture were performed throughout the study (**Figure 2B**). After the treatment, the patient visited every week in the following 2 months. Urine cultures and blood tests were obtained on each visit.

## Results

### Morphological Characteristics and Genome Sequence of Bacteriophages

**Figure 3:**
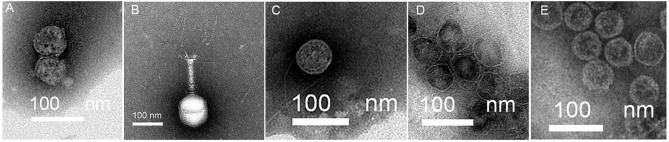
Transmission electron microscopy image of phages. **(A)** phage ФJD902. **(B)** phage ФJD905. **(C)** phage ФJD907. **(D)** phage Ф JD908. **(E)**, phage ФJD910.

Bacteriophage was screened against successive patient isolates as those isolates became available. A total of five bacteriophages Ф902, ФJD905, ФJD907, ФJD908, and ФJD910 were selected for therapy. Photograph of five bacteriophages was obtained by transmission electron microscopy, as shown in **Figure 3**. Morphologically, Ф902, ФJD907, ФJD908, and ФJD910 belong to the podoviridae family while ФJD905 belong to the myoviridae family. Ф902, ФJD907, ФJD908, and ФJD910 have genome sizes of 43,274, 39,465, 40,777, and 38,834 bp, respectively. ФJD905 has genome sizes of 147,174 bp. These five bacteriophages encoded ORFs were searched by blast in the database of virulence and antibiotic resistance genes (http://www.genomicepidemiology.org/), with no identifiable virulence or antibiotic resistance genes found in their genome.

### Heterogeneity of Bacterial Isolates

**Figure 4:**
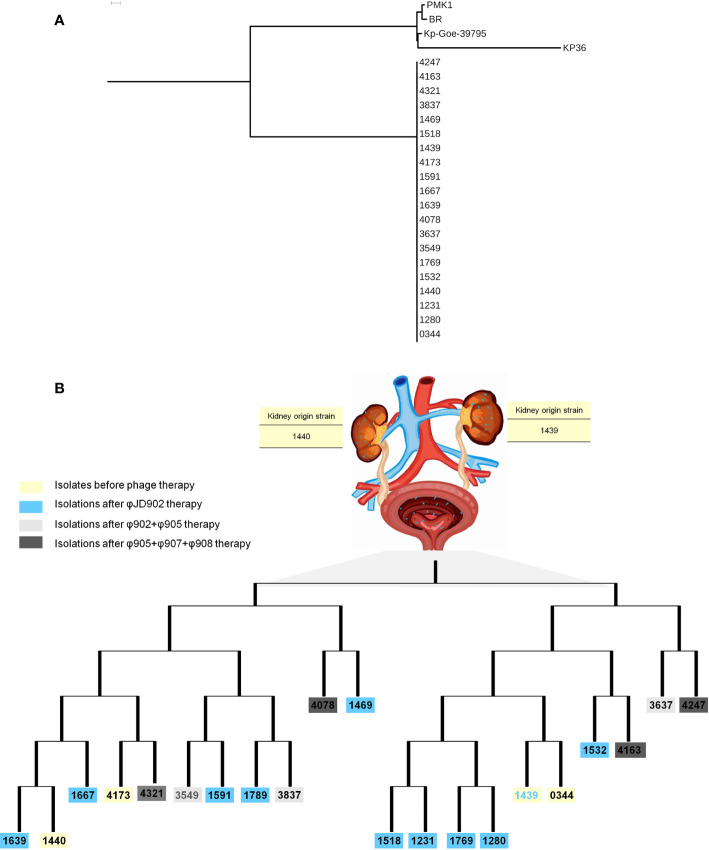
Phylogenetic trees of *K. pneumoniae* isolates. **(A)** The genome-wide SNP-based phylogenetic trees of 21 sequenced *K. pneumoniae* ST15 isolates and 4 completely sequenced ST15 *K. pneumoniae* isolates currently available at GenBank (PMK1, BR, Kp-Geo-39795, and Kp36). **(B)** The genome-wide SNP-based phylogenetic trees of 21 sequenced *K. pneumoniae* isolates by this study. The phylogeny scheme based on parsimony was generated from 9,170 SNPs for all genomes and 2,795 SNPs for 21 genomes by this study using kSNP3 with k = 21 and displayed by iTOL with midpoint rooting.

**Figure 5:**
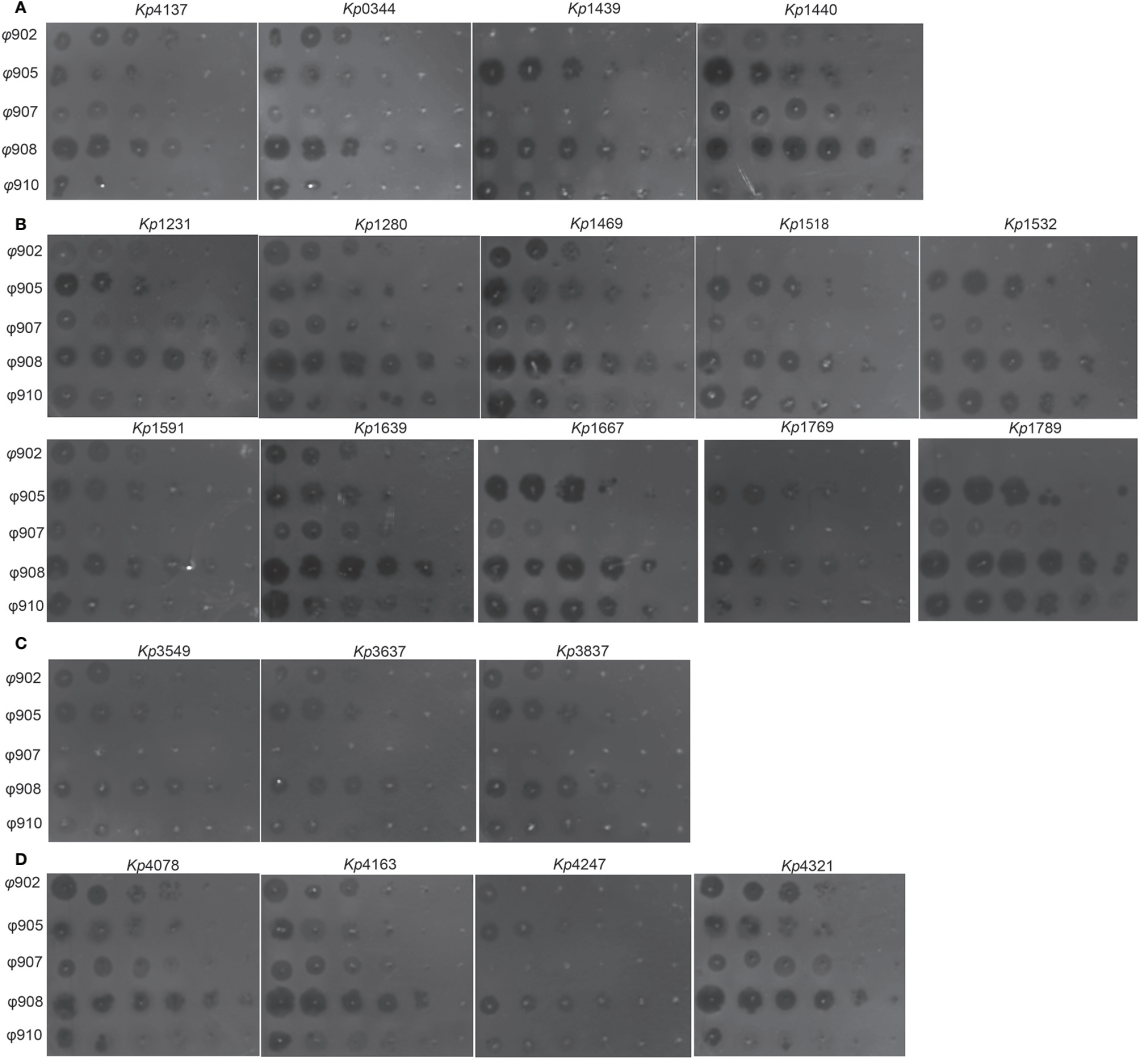
Phage susceptibilities of clinical isolates. **(A)** *K. pneumoniae* strains isolated before phage therapy. **(B)** *K. pneumoniae* strains isolated post ФJD902 therapy. **(C)** *K. pneumoniae* strains isolated post Ф902+Ф905 therapy. **(D)** *K. pneumoniae* strains isolated post Ф905+Ф907+Ф908 therapy. Phages were serially diluted 10-fold and spotted onto 21 *K. pneumoniae* as indicated. These assays were repeated three times with similar results and a representative experiment is shown.

A total of 21 K*. pneumoniae* strains were isolated from the patients during treatment. Whole genome sequence analysis showed that they all belong to ST15. The genome-wide detection of these 21 sequenced isolates and 4 completely sequenced ST15 *K. pneumoniae* currently available at GenBank (PMK1, BR, Kp-Geo-39795, and Kp36) generated 9,170 SNPs. The SNP-based phylogenetic tree analysis showed that these 21 isolates are from the same clone (**Figure 4A**). Further genome-wide detection of these 21 isolates generated 2,795 SNPs. The SNPs-based phylogenetic tree analysis showed the polyclonal strains (Kp4137, Kp0344, Kp1439, Kp1440) cloned in the patient before phage treatment (**Figure 4B**). The sensitivity of these strains to the phage 902, ФJD905, ФJD907, ФJD908, and ФJD910 was also different (**Figure 5A**). These results suggested that the patient was infected with polyclonal *K. pneumonia* for long time. These results suggest that the colonized *K. pneumoniae* has evolved into polyclone as a result of the patient’s chronic infection.

*K. pneumoniae* continued to be isolated in the urine of patients during bacteriophage ФJD902 treatment (**Table 1** and **Figure 2B**). Analysis of phage lytic spectrum of these 10 successively isolates showed that five strains (Kp1231, Kp1280, Kp1469, Kp1591, and Kp1639) were still sensitive to ФJD902, whereas the remaining five strains (Kp1518, Kp1639, Kp1667, Kp1769, Kp1789) developed resistance to ФJD902 (**Figure 5B**). Phylogenetic tree analysis showed that the five bacteriophage-sensitive strains are located in different branches. Similarly, the five bacteriophage-resistant strains are in different branches. It has been reported that bacteria mutate rapidly to develop phage resistance when a bacteriophage infects a bacterium (Hesse et al., 2020). It was speculated that the five bacteriophage-resistant strains may come from the mutation during bacteriophage therapy. However, it cannot be ruled out that it may be the heterogeneous strain colonized in the patient.

By screening for lytic bacteriophages against previous isolates, a two-phage cocktail (ФJD902+ФJD905) and a three-phage cocktail (ФJD905+ФJD907+ФJD908) was used to the continued treatment. However, after the phage cocktail was administered, the patient still had *K. pneumoniae* in his urine. As shown in **Figures 5C**, **D**, these isolates are still sensitive to the phage cocktail, suggesting that the phage cocktail did not function in some degree. It is speculated that heterogeneous bacteria colonized in the renal pelvis cannot be effectively cleared because the phage cannot reach them.

### Phage Therapy Outcome

According to the isolates (strain Kp4173 and Kp0344) from the patient urine, lytic phage ФJD902 against them was selected for his first therapy (**Figure 2A**). We performed a regimen of antibiotic withdrawal and a two-week phage administration by bladder irrigation. During the time, we placed a “Double J” stent to dredge the connection between the pelvis and bladder, and the phage was irrigated to the renal pelvis once during the placement process. Renal pelvis effusion was submitted to culture. Despite the ongoing phage therapy, urine cultures were positive (**Figure 2B**). Five isolates recovered from urine developed resistance to ФJD902 (**Figure 3B**). Bilateral pelvis effusion cultures were positive.

Thus, a phage cocktail containing ФJD902 and ФJD905 lytic to all previous isolates was administrated for second therapy *via* bladder irrigation. The patient felt relief of his symptoms with negative urine culture during therapy. Considering that the double J stent was in place for two months, we felt it necessary to replace it with a new one (**Figure 2A**). The right stent was successfully removed, while the left stent was missing. Unexpectedly, his urine culture became positive again (**Figures 2B**, **3C**). Following screening, an adapted phage cocktail containing ФJD905, ФJD907, and ФJD908 for the third therapy *via* bladder irrigation were continued. However, the patient’s urine culture remained positive with *K. pneumoniae*. We had to halt the phage therapy and replaced it with antimicrobial therapy with piperacillin/tazobactam (**Figure 2A**).

According to the renal pelvis culture*, K. pneumoniae* colonized the kidney. We hypothesized that the heterogeneous pathogens in the renal pelvis were unreachable by phage cocktails *via* bladder irrigation. Thus, they could be released to the bladder continuously. To remove pathogens colonizing the bladder and renal pelvis, phage should be irrigated both *via* kidney and bladder. After obtaining informed consent from the patient, we performed PCN on the patient prior to phage therapy (**Figure 2A** and **Figure S1**). A phage cocktail containing ФJD902, ФJD905, ФJD908, and ФJD910 was irrigated *via* the pelvises and subsequently the bladder (**Figure 2A**). At the same time, the administration of piperacillin/tazobactam continued to enhance the eradication of minority subpopulations of phage-resistance variants. Then, phage therapy continued for another 10 days without antibiotic treatment (**Figure 2A**). Finally, the patient recovered with an obviously improved bladder with smooth mucosa (**Figure 1B**). MDR *K. pneumonia* infection did not recur after two months of follow-up as determined by culture growth from the patient’s urine.

## Discussion

Although UTIs are normally not considered life-threatening, these recalcitrant infections lead to unbearable symptoms of urinary irritation and diminished quality of life (Portsmouth et al., 2018). The bactericidal mechanism of bacteriophages is completely different from that of antibiotics. When bacteriophage therapy is administered, it is necessary to screen for highly lytic activity bacteriophages directly to the bacterial pathogen that is causing a clinically relevant infection (Schooley et al., 2017; Corbellino et al., 2019; Dedrick et al., 2019). Therefore, the clinical isolates are of great significance for guiding clinicians in choosing optimal bacteriophage therapy. Bacterial heterogeneity means that a patient may have polyclone bacterial infections, and generally we can only diagnose the main population and miss the subpopulations with low densities (Andersson et al., 2019; Nicoloff et al., 2019).

In this case, heterogeneous *K. pneumoniae* colonized renal pelvis and bladder prior to phage therapy. However, for our first phage therapy, we were able to isolate *K. pneumoniae* strain only from the urine and performed phage screening against these isolates. The patient’s urine culture still had phage sensitive and phage-resistant strains during phage therapy. It was later discovered that isolates from the renal pelvis and the bladder were different clones, and that *K. pneumoniae* colonized in the renal pelvis was not sensitive to therapeutic bacteriophage ФJD902. Even with the phage cocktail treatment, phage-resistant strains were rapidly isolated from the patient’s urine. These resistant strains could be the result of minor undetected resistant populations or from mutations. In this case, the patient’s renal pelvis is also colonized by pathogenic bacteria, which makes it difficult for phages to reach *via* bladder irrigation. We speculate that these are the two main causes of treatment failures.

In the following treatment, we performed a PCN on the patient so that phages could be irrigated *via* the kidney. A cocktail of bacteriophages was selected for activity against all previously isolates and irrigated simultaneously *via* the kidney and bladder, using antibiotics in combination. It is reported that bacterial mutation to bacteriophage resistance has also been associated with significant fitness costs of the reduction of antibiotic resistance or virulence (Ofir and Sorek, 2018; Gordillo Altamirano and Barr, 2019). Therefore, we hypothesized that the eventual successful clearance of *K. pneumoniae* was due to the synergistic effect of bacteriophage and antibiotics.

## Conclusion

In recent years, phage is expected as potential effective therapeutic agents for the untreatable infections (Gordillo Altamirano and Barr, 2019; Monteiro et al., 2019; Schmidt, 2019). In this case, as a patient with urinary tract infection, we designed phage treatment *via* bladder effusion for first therapy since it was easy to perform and less invasive. However, *K. pneumoniae* infection did not clear due to bacterial colonization of the renal pelvis and bacterial heterogeneity. Thus, we performed simultaneous bladder and renal pelvis perfusion using bacteriophage cocktails with activity against a range of pathogen. We developed a personalized approach for the patient, including phage cocktail-made and administration. The success of this case provides valuable ideas and solutions for personalized phage therapy of complex infection.

## Data Availability Statement

The datasets presented in this study can be found in online repositories. The names of the repository/repositories and accession number(s) can be found in the article/**Supplementary Material**.

## Ethics Statement

This trial was registered at the Chinese Clinical Trial Registry (www.chictr.org.cn) (ChiCTR1900020989). The patients/participants provided their written informed consent to participate in this study.

## Author Contributions

JQ, XG, TZ, JB, NW, and XS contributed to the study design. JQ, JB, NW, XS, SY, WZ, ZW, JC, LL, MG, JW, HL, DT, JZ, QH, ZZ, YS, and CH contributed to participant recruitment and data collection. JQ, NW, XS, and HO did the data analyses. JQ, NW, JB, XG, and TZ wrote the manuscript. All authors contributed to the article and approved the submitted version.

## Funding

This work was supported by Natural Science Foundation of Shanghai under Grant 17ZR1415900; Shanghai Public Health Clinical Center under Grant SJTNY. National Major Science and Technology Projects of China under Grant 2020ZX09201001-005-003; Shanghai Hospital Development Center under Grant SHDC2020CR2028B.

## Conflict of Interest

The authors declare that the research was conducted in the absence of any commercial or financial relationships that could be construed as a potential conflict of interest.
