# Supplementary figures and images for: Heterogeneous Klebsiella pneumoniae Co-infections Complicate Personalized Bacteriophage Therapy
Source: Front Cell Infect Microbiol. 2021 Jan 25;10:608402. doi: 10.3389/fcimb.2020.608402 (PMC7868542; doi:10.3389/fcimb.2020.608402)

**Supplementary Figure**

**
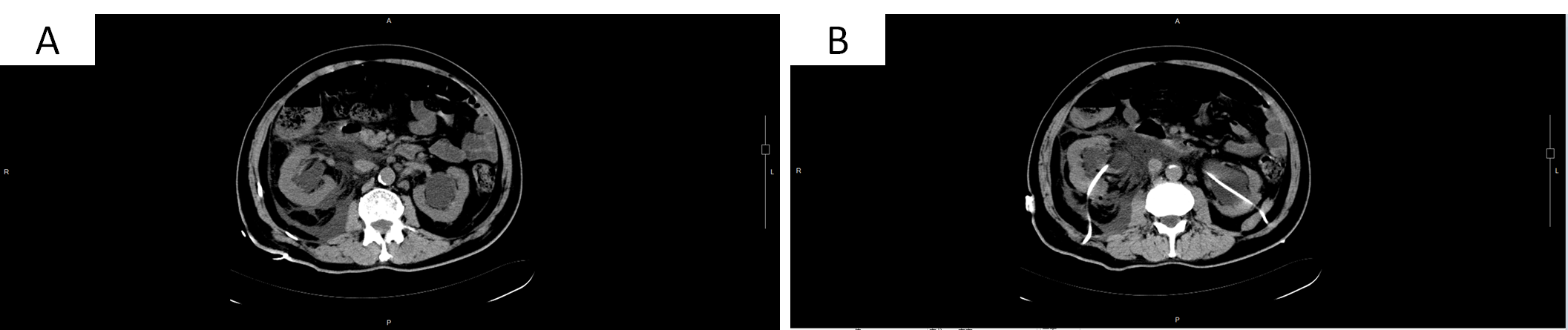
**

**Figure S1.** CT scans of the kidneys without (A) and with (B) PCN

Supplement: Supplementary file 1 [file DataSheet_1.docx]
